# Enhanced RF Behavior Multi-Layer Thermal Insulation

**DOI:** 10.1038/s41598-017-18337-3

**Published:** 2018-01-08

**Authors:** A. Mazzinghi, M. Sabbadini, A. Freni

**Affiliations:** 1 DINFO, University of Florence, Florence, Italy; 2 European Space Agency, Noordwijk, The Netherlands

## Abstract

This paper shows that it is possible to exploit the modulated metasurface concept to control the unwanted coupling between antennas that are installed on the same satellite. The metasurface is combined with a Multi-Layer thermal Insulation blanket to reduce its specular reflection by spreading the energy incoherently in the surrounding space. In the design, sub-wavelength radiating elements printed on thin substrate have been used to make the metasurface response azimuthally independent, and to keep the weight of blanket down. The comparison between simulations and measurements confirms the validity of the idea.

## Introduction

Multi-layer thermal Insulation (MLI) blankets are usually employed in communication satellites and other spacecrafts for shielding electrical and electronic componentry from the space environment through which the spacecraft travels. Due to their high reflectivity, MLI blankets can contribute to unwanted coupling between antennas set on the same platform. Consequently, cross-coupling among adjacent antennas, which is often required to be lower than −70 dB^1^, can reach inappropriate levels.

In this paper, we demonstrate that it is possible to exploit the modulated metasurface concept^2^ to help controlling antenna coupling. Metasurfaces are thin metamaterials constituted by periodic small elements “where the period of the structure is still small compared to a wavelength, but the individual scatterers are designed in such a manner (either via their shape or their constitutive properties) that the scatterers themselves can resonate”^3^. Modulated metasurfaces are metasurfaces where the element geometry and size are changed locally to control the scattered wave.

To reduce the coupling effect between antennas on board of the same platform, metasurfaces can be used either as an absorber or as a scatterer. The former solution can however influence the thermal properties of the blanket, increasing its temperature^4^. Thus, thermal insulation requirements can become more unfavorable to be satisfied. Here, a modulated metasurface, printed on top of the MLI, is employed to reduce the field scattered in the direction of maximum antenna coupling, by spreading the impinging electromagnetic wave everywhere. In particular, the metasurface is designed to scatter the impinging field almost isotropically, independently of the direction of arrival, which is not exactly known *a priori*. Indeed, the position and the shape of the MLI are set only when the satellite is assembled, and can differ significantly from what was specified in the stage of planning. The proposed solution minimizes the impact on the thermal design of the blanket, while significantly influences its radio frequency (RF) response. Furthermore, the choice of a metasurface as scatterer instead of as an absorber, allows the use of thin substrates, thus preserving the MLI flexibility and weight.

Recently, metasurfaces have been proposed to work as lenses for shaping the beam generated by a simple source^5–7^. A few authors have also proposed to print sub-wavelength metallic elements on an extra dielectric layer on top of a coupling surface to reduce its radar cross section (RCS)^8–12^. In particular, they suggest distributing, in a periodic lattice, sub-wavelength elements whose dimensions are chosen at random in a specific range. This has been done with the false convincement that each radiating element introduces a phase variation between the incident and the scattered field equal to the one calculated with the local periodicity approximation^13^. However, since the radiating elements are sub-wavelength, a strong mutual coupling exists between adjacent elements (i.e. elements whose distance is less than half a wavelength). This means that, when close elements are significantly different, as for example in a random distribution, the phase variation introduced by a single element is strongly influenced by the adjacent ones. Therefore, the final behavior of the metasurface designed as in^8–10^ is not predictable by using the local periodicity approximation, as will be demonstrated in Sec. III. Moreover, when half a wavelength period is used, as in^9^, the reflection coefficient associated to each element is no more independent from the azimuthal incidence angle. Thus, the metasurface behavior becomes anisotropic.

In this paper, a strategy for distributing sub-wavelength elements along the metasurface is proposed. It differs from what suggested in the previous papers since it enforces that the phase delay, that each element provides along the metasurface, changes in a continuous manner, and does not vary faster than 360° if one covers a distance of one free space wavelength.

Contrary to classical radar applications, here the strict requirements on MLI usable materials and thickness have been considered. Specifically, only Kapton, Mylar, or Teflon (common materials employed in thermal blankets) can be used, and the metasurface must have a small thickness (preferably 0.5 mm or less), so as to preserve its flexibility and low weight.

The introduction of the metasurface on top of the blanket does not alter its manufacturing complexity, since it makes use of the same manufacturing technology of the unloaded thermal blankets. When a thin substrate is used, as in this case, the required 360° phase-delay range can be easily covered even by sub-wavelength radiating elements. However, this phase variation takes place in a very small change of the elements dimension. For example, for a square frame element the 360° cycle occurs at 16.6 GHz in the range of 3.5 ÷ 4.5 mm. The latter property introduces high sensitivity of the metasurface to the manufacturing tolerances, and an enhanced sensitivity to the thermal expansion. However, these effects play a minor role in a randomized surface as the one presented here.

Two other issues need to be addressed to make the concept applicable to satellites: charging and passive inter-modulations. However, both are common with standard thermal blankets and they can be taken into account as it is usually done in the design for flight-qualified items.

## Scenario

**Figure 1:**
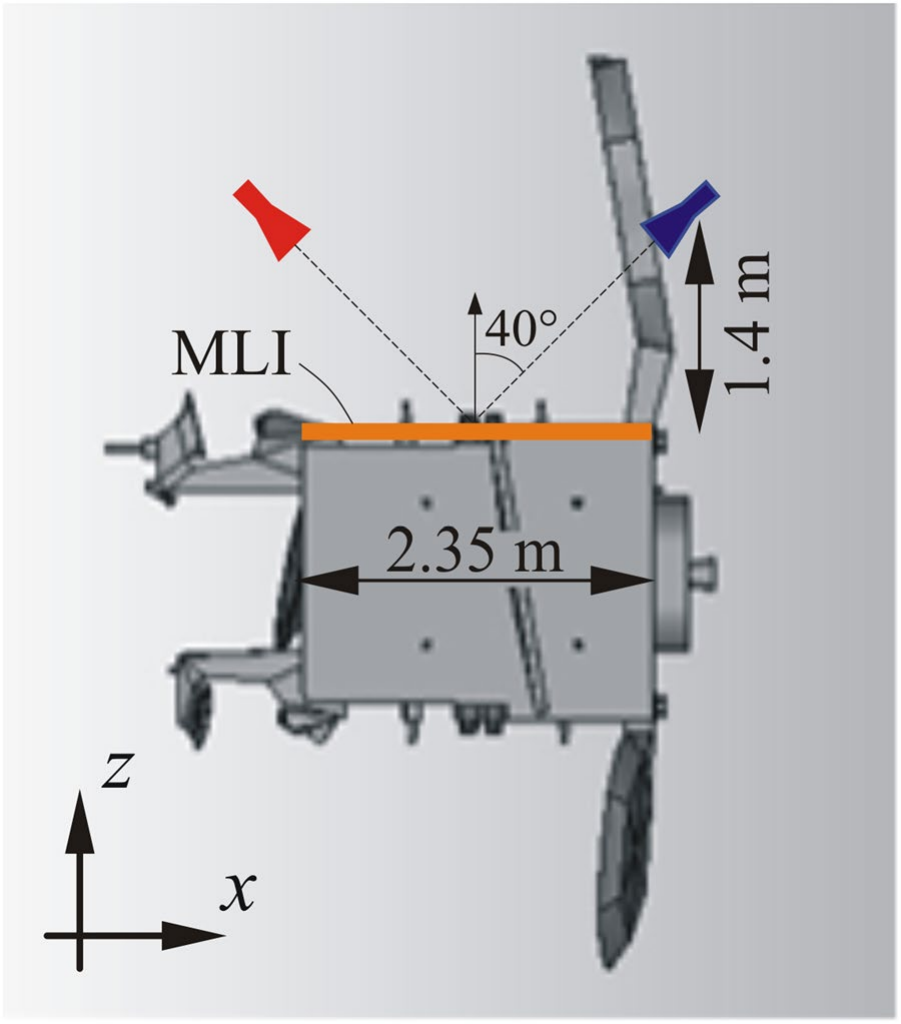
Reference satellite scenario: the actual geometry is simplified as a transmitting antenna plus a receiving one located over a satellite flat face (2.3 m in the longitudinal direction, and 1.7 m in the transversal one) covered with a metasurface MLI. The transmitting horn (red colored) is a Ku 20 dBi standard horn, 40° tilted with respect to the satellite face. The receiving 15 dBi gain horn (blue colored) is located symmetrically to the transmitting one.

A typical satellite scenario is here considered to evaluate the effectiveness of the metasurface MLI by reducing the antenna coupling. Two antennas working in Ku band are located over the opposite sides of a satellite face, which is covered with a planar thermal blanket, as sketched in Fig. 1. Specifically, a transmitting *y*-polarized standard horn (characterized by 20 dBi gain and with a working band of 11.9–18 GHz) illuminates the thermal blanket with an angle of 50° with respect to the satellite surface normal vector. The receiving antenna, characterized by a *cos* ^7.5^(*θ*) pattern (i.e., a directivity of about 15 dBi), is located symmetrically with respect to the transmitting one. This antenna configuration is certainly unrealistic, but it represents the worst-case for the analysis of an on-board antenna coupling.

Since in real environment the exact geometry of the MLI cannot be known *a priori*, the direction and the polarization of the impinging field on the metasurface are not well predictable. Thus, the metasurface MLI should behave similarly for all possible impinging directions, and for both TE and TM polarizations. The latter characteristic can be easily achieved by using sub-wavelength elements. This choice also makes the element response independent on the azimuthal incidence angle of the impinging wave^14^.

## Design Strategy

Different synthesis approaches can be adopted to reduce the antenna coupling. For example, a reflectarray-like approach, that exploits a tailored metasurface as large as the entire thermal blanket, could be used to create a null in a specific desired direction. However, this method is not applicable here since both the position and the shape (i.e., the deviation from the planar configuration) of the MLI in its operational environment is only approximately known *a priori*. Thus, a small variation from the nominal set up would lead the null in an uncontrolled position. Furthermore, the side lobes introduced by the metasurface should be controlled as well, so as not to influence other adjacent antennas^15^. These drawbacks suggest us to distribute the scattered power in the space as uniformly as possible for a wide range of elevation incidence angle, independently from the azimuthal one.

The simplest solution is to choose the dimension of each element randomly, either along the entire metasurface^10^, or in a macro cell which is periodically repeated to cover the MLI^11,16,17^. However, this solution is not feasible when sub-wavelength elements are employed in order to assure the independency of the scattered field from the azimuthal direction of incidence. In fact, the phase delay introduced by adjacent sub-wavelength elements can only differ slightly.

**Figure 2:**
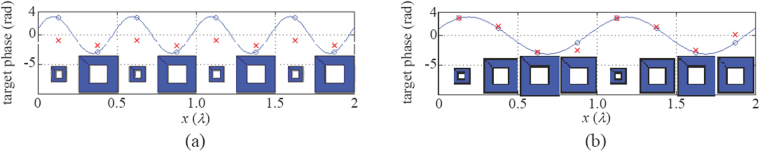
Comparison of the target phase (blue dots) and the actual phase (red crosses) delay introduced by the different cell element distributions when the size variation has a period equal to: (**a**) *λ*/2 and (**b**) *λ*.

**Figure 3:**
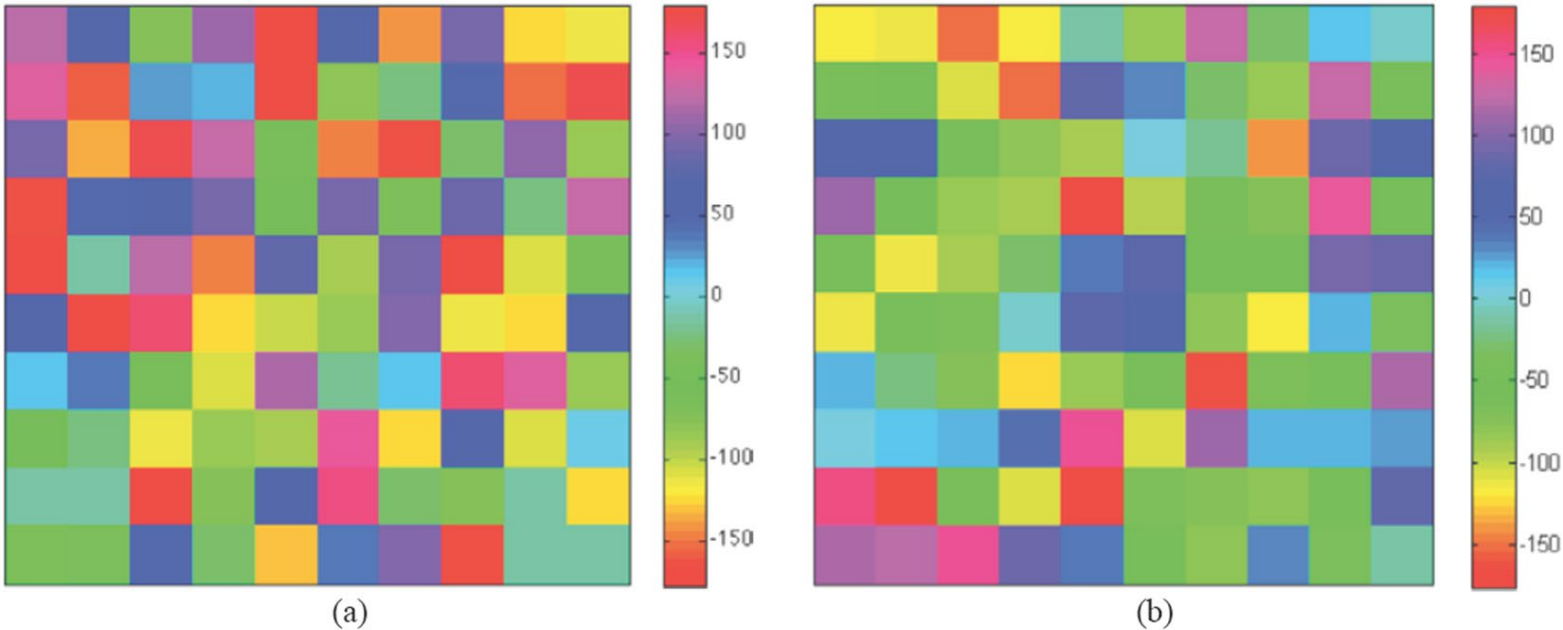
Phase variation along a 10 × 10 pixels macrocell: (**a**) when the local periodicity approximation is adopted and (**b**) when a full wave analysis is performed. The shown macrocell is the basic cell of a periodic metasurface.

To explain this concept better, let us consider the two simple examples of Fig. 2, where sub-wavelength elements, λ/4 apart, are employed. Figure 2a is relevant to what we can call a fast phase variation: the elements have to introduce a phase delay between incident and scattered field of almost 180° along a distance shorter by λ/2. Figure 2b concerns what we call a slow phase variation, i.e. a phase delay less than 180° along a distance equal to λ/2. In both figures, the blue curve represents the target phase variation that the metasurface has to provide. The blue dots are the phases that each element (shown in the inset) should introduce when it is considered in a periodic lattice of equal elements. It is worth noting that this corresponds to the “local periodicity approximation”^13^ commonly used in the reflectarray design^18^, and that has been also employed in^8–12^ for the metasurface design. The red crosses show the actual phases realized by each element when the entire metasurface is analyzed by a full wave method^19^ that can be regarded as the exact solution. It is evident that, when element size modulations faster than half a wavelength are considered (i.e. a fast phase variation), the strong coupling between adjacent elements significantly modifies the phase delay calculated by using the local periodicity approximation. This effect is even more evident if a random phase is synthetized on a 10 × 10 elements macrocell as in^11^. Starting from the target reflection phase distribution shown in Fig. 3a, the layout of the metasurface has been designed according to the local periodicity approximation, when the element shape in the inset of Fig. 2 is used. Figure 3b shows the actual phases introduced by each element, when calculated by a full wave analysis of the entire metasurface. Clearly, there is no agreement between the two phase maps; so, also the associated radiation patterns will be completely different. This does not mean that the approach in^8–12^ would not determine a reduction of the scattered field in the specular direction, even if the phase distribution will not be completely random, as expected, but it will result in a haphazardly manner due to the neglected strong inter-element coupling. However, the actual metasurface response in the whole space will not be predictable and only a full wave simulation of the entire structure, or its measurement, will provide a true description of the scattering. The non-predictability of the scattered field is even worse for our specific task. In actual fact, by using the local periodicity approximation the presence of grating lobes cannot be correctly described. These are not important when the only issue is the RCS, but their presence can be really unfavorable in a complex environment as a satellite is.

A slow variation of the dimension of adjacent sub-wavelength elements (i.e., a slow phase variation) is also mandatory to guarantee a good frequency stability of the metasurface characteristics. In fact, in this case, the relative phase variation between adjacent elements will not be drastically modified by changing the frequency.

Here, the metasurface is split up into tiles where a slow phase variation between adjacent elements is assured. Each tile is commensurate to the others and it is designed to generate an omnidirectional broad beam scattering pattern. Nearby tiles generate the same pattern beam, but their relevant scattered fields differ for a random phase, so as to generate incoherent scattering contributions. In the next subsections, the design strategy is described in detail.

### Design of the Metasurface Tile

The tile dimension has been chosen as a compromise between beam shaping and phase delay that each element in the tile has to introduce. In fact, a small tile dimension, but however larger than a wavelength, does not provide enough degrees of freedom to accurately synthetize in the space the desired scattered field distribution, which has to be as isotropic as possible. Moreover, the tile dimension (i.e., the tile repetition period) will determine the number and position of the grating lobes in the visible spectrum^20^.

Since the relative position between the reflection point on the metasurface, and the transmitting and receiving antennas are known only approximately, the tiles dimension has also to be small enough to consider the impinging field with uniform amplitude and linear phase distribution, independently on the tile location. This means that both the source and the observation points have to be in the far field of each tile. However, since the total field is the sum of almost incoherent contributions from all the tiles, as it is in optics, it is sufficient to satisfy the condition *r* > *D* ^2^/*λ*, where *r* is the distance of the source/observation point from the tile centre, and *D* is the maximum linear dimension of the tile. For example, in the reference scenario of Fig. 1 all the tiles are at least 1.4 m from the antennas. Thus, each tile has to be chosen smaller than 6.2λ × 6.2λ.

**Figure 4:**
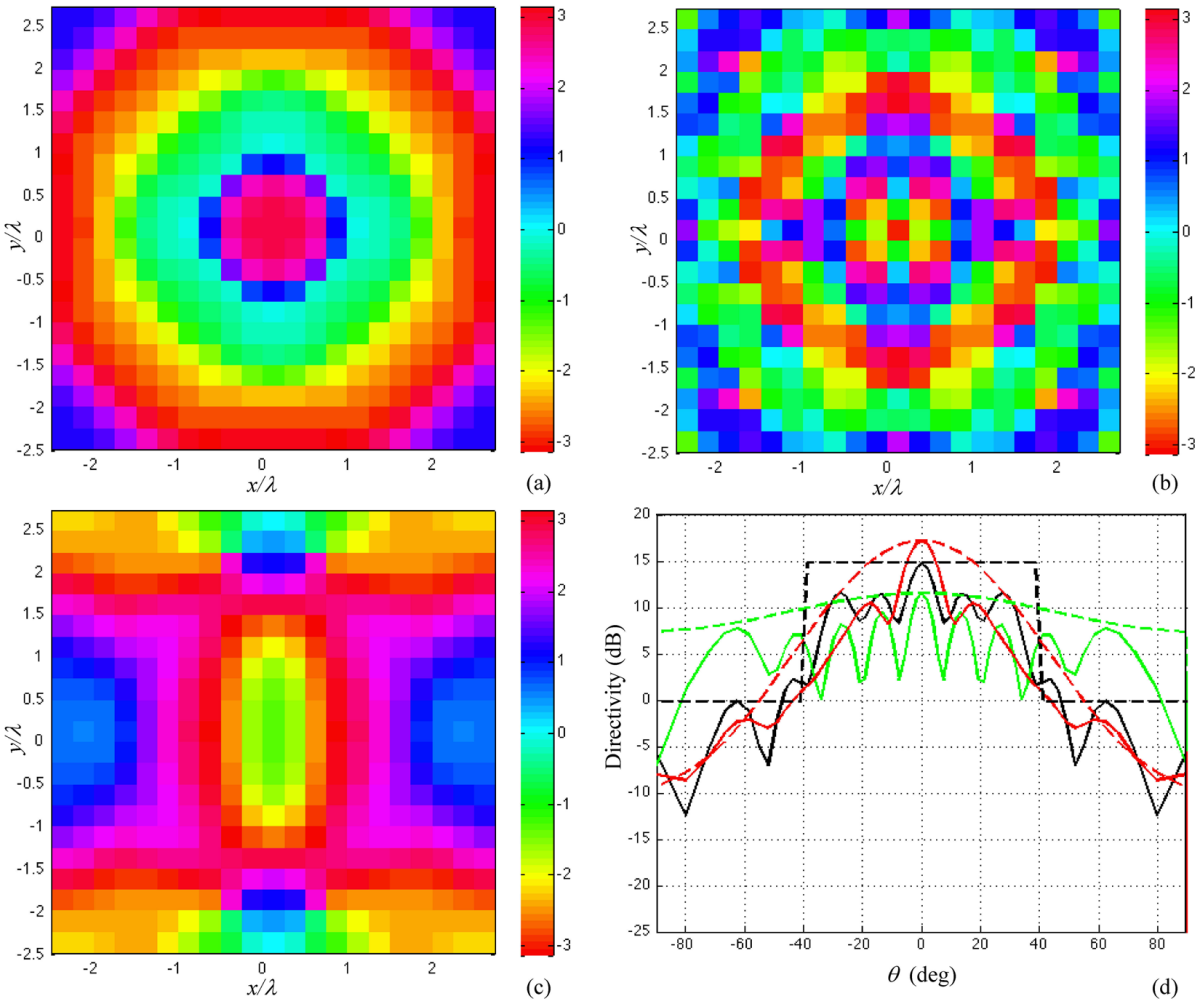
Phase distribution map obtained by imposing (**a**) a broad beam mask (80° BW), (**b**) a guassian beam mask with 60° BW, (**c**) a gaussian beam mask with 20° BW. (**d**) shows the comparison between the synthetized beams and the relevant optimization masks.

For our reference scenario, from a comparative analysis not reported here for the sake of compactness, a 5λ × 5λ tile results in the best compromise. Then, the phase delay distribution on each tile has to be determined so that the impinging field is scattered as uniform as possible in the surrounding space. Here, starting from the desired shape of the scattered field, we calculate the corresponding tile phase map by using a 2D Alternate Projection Method^21^. Figure 4 shows the phase delay distribution on the tile, provided by the method, for three different beam shape targets, plus the relevant synthetized beams. Among the different solutions, the one obtained by imposing a broad beam mask (black curves in Fig. 4d) appears to be the most valid since a smooth spatial phase delay variation is obtained (see Fig. 4a). In addition, Fig. 4a shows a convenient axial symmetry that assures a good independence from the azimuthal direction of the impinging wave.

It is worth noting that, due to the limited tile dimensions, in all the considered cases, the shaped beam obtained as output of the optimization procedure is only approximately equal to the imposed pattern mask. The evident oscillating behavior, however, does not represent a drawback for the final design goal of spreading the scattering field everywhere.

### Metasurface Tile Repetition

The entire metasurface is obtained by repeating periodically the basic tile *M* times along the *x* axis and *N* times along the *y* axis. The phase delay associated to each element (*i*, *j*) in the basic tile is denoted *ϕ*_*i*,*j*_. The relative phase delay between two elements belonging to the same (*m*, *n*) tile is independent from the tile position in the metasurface. The phase delay introduced by the element (*i*, *j*) in the tile (*m*, *n*) is given by

1 $${\phi }_{i,j}^{(m,n)}={\phi }_{i,j}+{\phi }^{(m,n)}$$

where *m *= 1, …, *M* and *n *= 1, …, *N*, and the tile global phase *φ* ^(*m*,*n*)^ is chosen at random. Thus, the phase introduced by elements in the same position in the tile, belonging to two different tiles, differs for the random phase *φ* ^(*m*,*n*)^. However, the phase difference between corresponding elements in two adjacent tiles is forced not to overtake ±*π*/2, i.e.

2 $$|{\phi }^{(m,n)}-{\phi }^{(m+{\rm{\Delta }}m,n+{\rm{\Delta }}n)}|\le \frac{\pi }{2},\,\,\,\,\,{\rm{\Delta }}m,{\rm{\Delta }}n=-1,0,+1$$

Moreover, to make the transition of the phase smooth between adjacent tiles, a Gaussian blur filter, with standard deviation equal to $1/\sqrt{\pi }$ (that results in a standard deviation of the transition equal to *π*/2), has been applied to the tiles in a region of a wavelength across the edges. The reduced phase range variation with respect to a completely random one assures that contiguous elements actually introduce the expected phase. Moreover, the removal of the any periodicity prevents the grating lobes arising.

**Figure 5:**
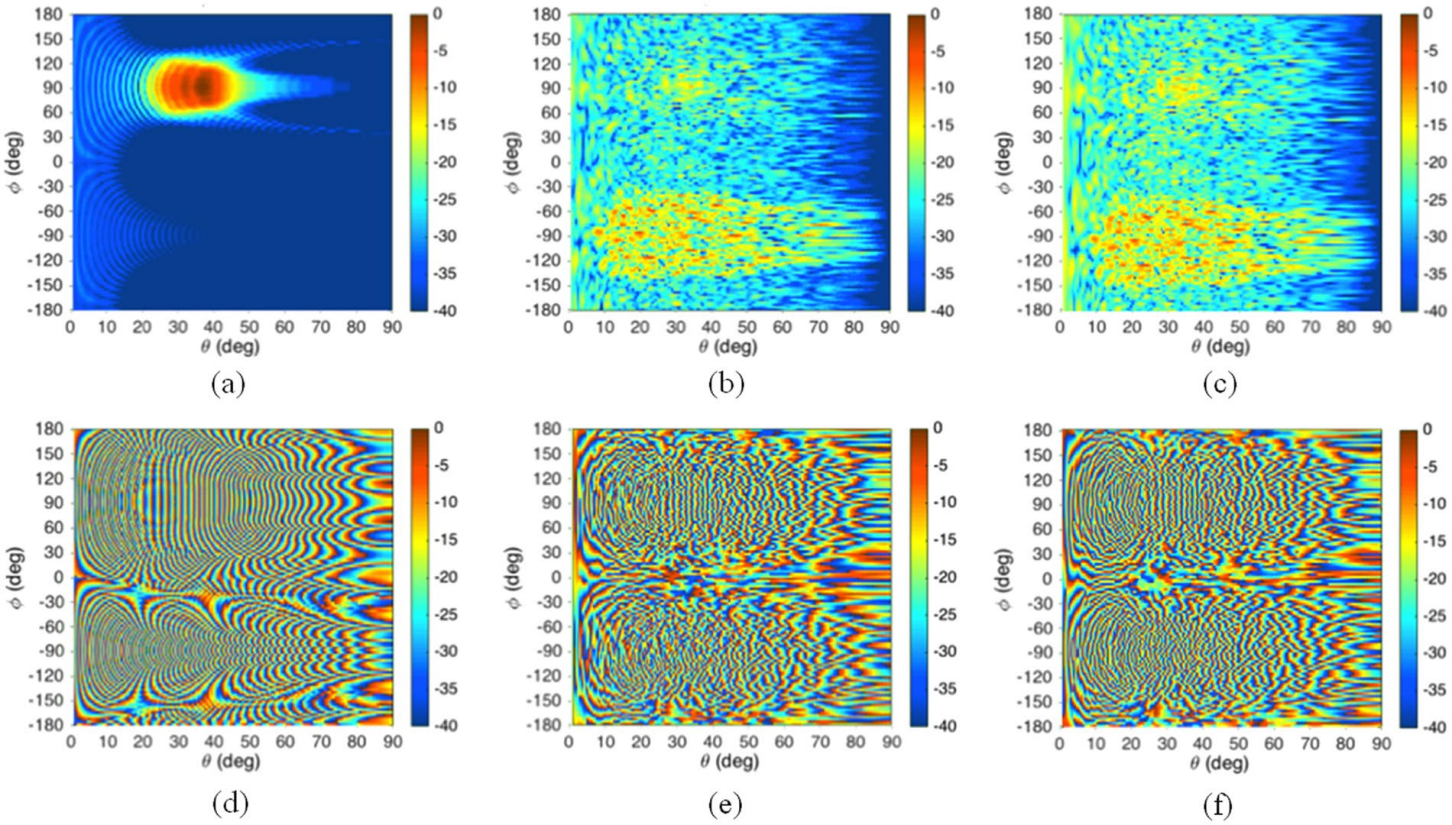
Power density (**a**–**c**) and phase distribution (**d**–**f**) of the scattered field in the whole space for a continuous metallic plate (**a**,**d**) or for the modulated metasurface with limited random phase, for the reference scenario of Fig. 1, when calculated by using the Physical Optics approach (**b**,**e**) or a full-wave fast technique (**c**,**f**).

The suggested approach allows us to obtain reliable results by simply using a Physical Optics approximation for calculating the field scattered by the entire metasurface, with a significant reduction of the computation time with respect to a full wave fast technique. This also allows evaluating the main performance of the metasurface even before choosing the element shape that will achieve the desired phase. For example, for the specific scenario here considered, Fig. 5 shows the power flux density and the phase of the field scattered all over the space in the case of the continuous metal plate and of the metasurface. It is worth noting that, not only the power flux density is reduced by more than 15 dB in the specular direction, but also the phase distribution of the field scattered by the metasurface is almost incoherent. This further reduces the actual coupling to any extended target. In particular, in the reference scenario of Fig. 1, the coupling between the two horns is reduced by 25 dB. This coupling has been evaluated by calculating the field contributions associated to each metasurface element in the receiving point and, from them, the open circuit voltage at the receiving antenna port.

To highlight the reliability of the Physical Optics approach, in Fig. 5c and f we also report the power flux density and the phase of the scattered field obtained by using a full wave fast technique^19^ for analyzing the entire metasurface, when the square frame element, described in the next section, is used. It is evident that the so obtained results almost perfectly match those calculated with the Physical Optics approach (Fig. 5b and e).

## Element Design

As soon as the phase delay that each element of the tile has to introduce is specified, the element dimensions can be calculated by using the local periodicity approximation^13^. The element shape choice is driven by the following requirements: ideally, the element should have the same phase response for both TE and TM polarizations when its geometry is changed, and it should cover at least 360° phase range, with a linear smooth variation with respect to its size. This is strongly opposed by the requirement on the small substrate thickness and by the sub-wavelength dimension of the pixel. Thus, a compromise has to be found.

**Figure 6:**
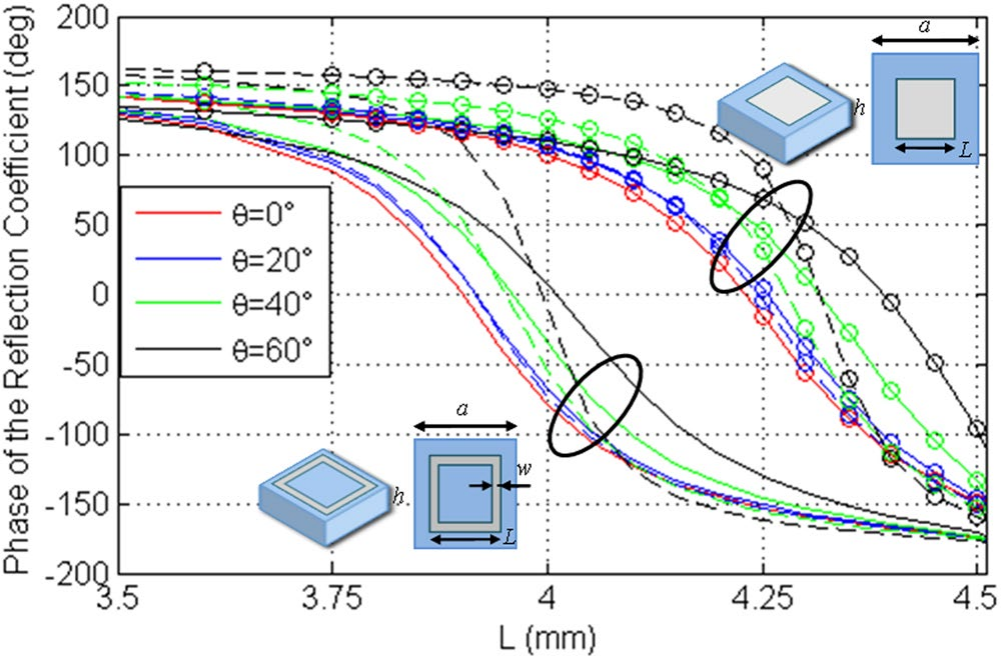
TE (continuous curves) and TM (dash curves) reflection coefficient phases at 16.6 GHz as a function of the element dimension for a few values of the incidence angle.

Figure 6 shows the reflection coefficient phase versus the element dimension, when the element is embedded in a periodic lattice of period *a *= *λ*/4 = 4.518 mm at 16.6 GHz, and a 0.5 mm thick layer of Mylar (*ε*_*r*_ = 2.8 − *j*0.0224) is chosen. In Fig. 6 the performance of a full square patch and a square frame with *w *= *L*/4 are compared. Even if the square frame is the best solution and it will be used in the final design, the full square patch has been analyzed because it allows a low cost manufacturing of a prototype large enough to demonstrate the concept. Since this element does not present small details, it allows the use of a vinyl cutting plotter and adhesive copper foil to manufacture the upper patterned metallic layer of the metasurface which can be simply stuck on the Mylar surface.

## Validation and Results

**Figure 7:**
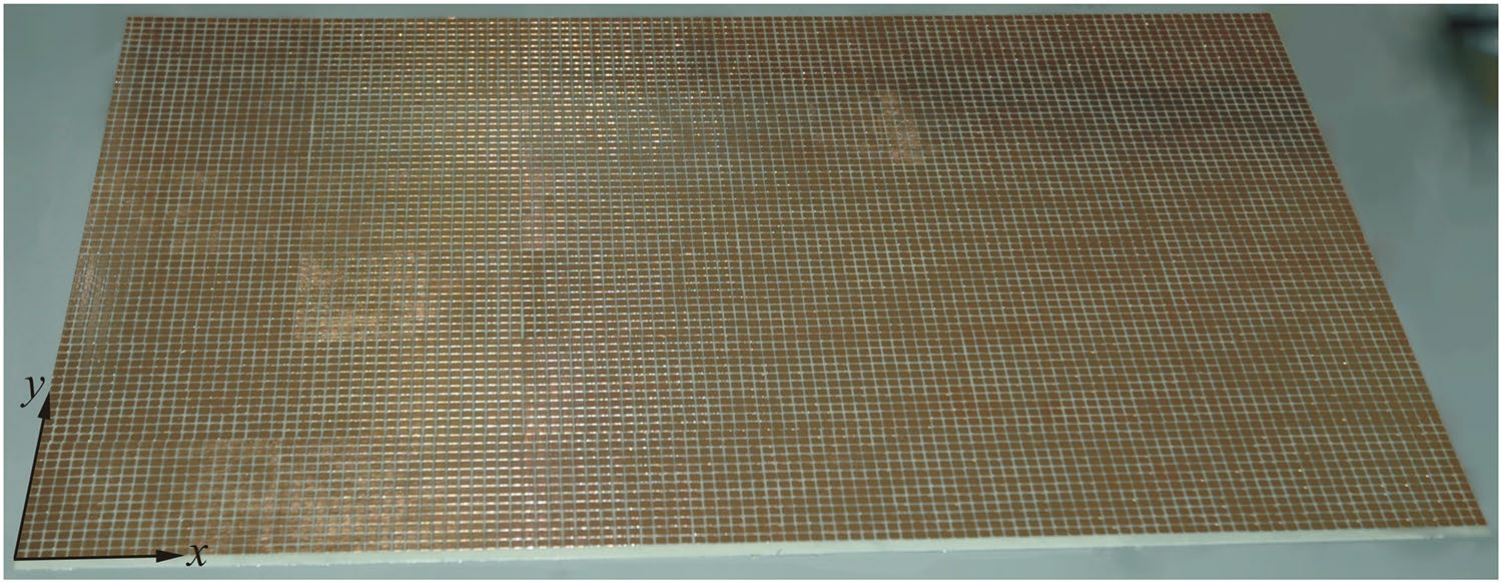
Photograph of the manufactured 60 × 40 cm^2^ metasurface.

**Figure 8:**
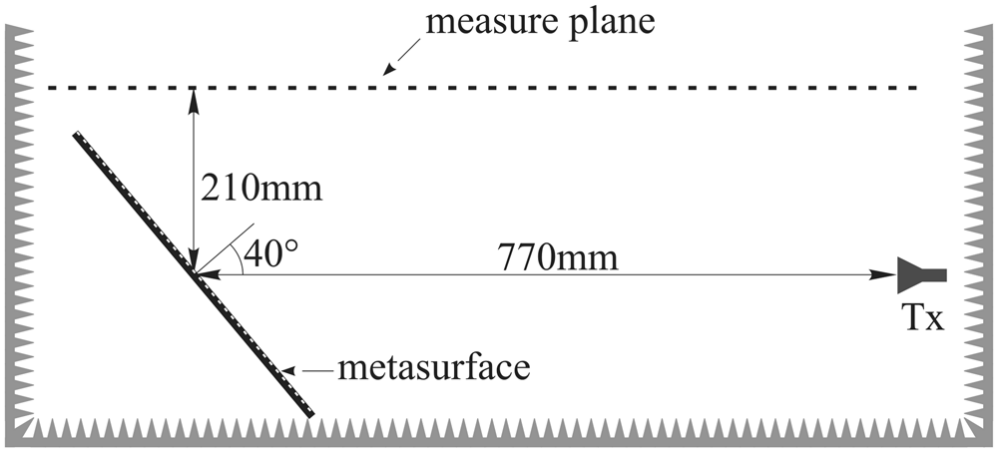
Near field measurement set-up.

To achieve a first low-cost validation of the concept, a “homemade” prototype (Fig. 7), has been manufactured. Specifically, a thermal blanket commonly used for sport applications (having electrical characteristics very similar to those of the thermal blankets for space applications) has been glued on a 0.5 mm thick layer of Mylar. Then, a 32 μm thick adhesive copper sheet was cut with a vinyl cutting plotter and stuck on the free face of the Mylar. Even with poorer performances than the square frame element, the full square patch element has been considered suitable to be manufactured with this technique. Figure 7 shows a photograph of the manufactured 60 × 40 cm^2^ metasurface integrated thermal blanket, which has been inserted in our near field scanner. Figure 8 shows the measurement set up adopted to acquire the scattered field on a plane at a distance of 210 mm to the metasurface center, whose unit normal vector is tilted by 40° with respect to the metasurface surface.

**Figure 9:**
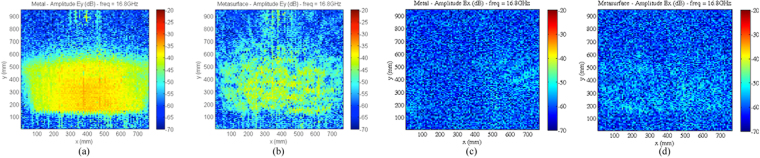
Amplitude map of the co-pol. (**a**,**b**) and cross-pol. (**c**,**d**) measured field at 16.8 GHz for the continuous metallic plane (**a**,**c**) and for the metasurface (**b**,**d**), when the MLI is flat.

**Figure 10:**
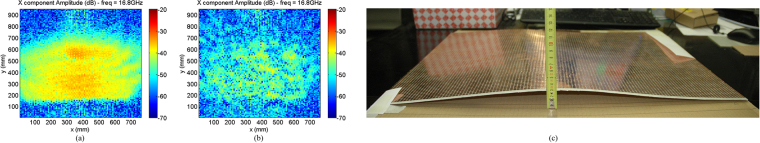
Amplitude map of the co-pol. (**a**,**b**) measured field at 16.8 GHz for the continuous metallic plane (**a**) and for the metasurface (**b**), when the MLI is deformed as in (**c**).

**Figure 11:**
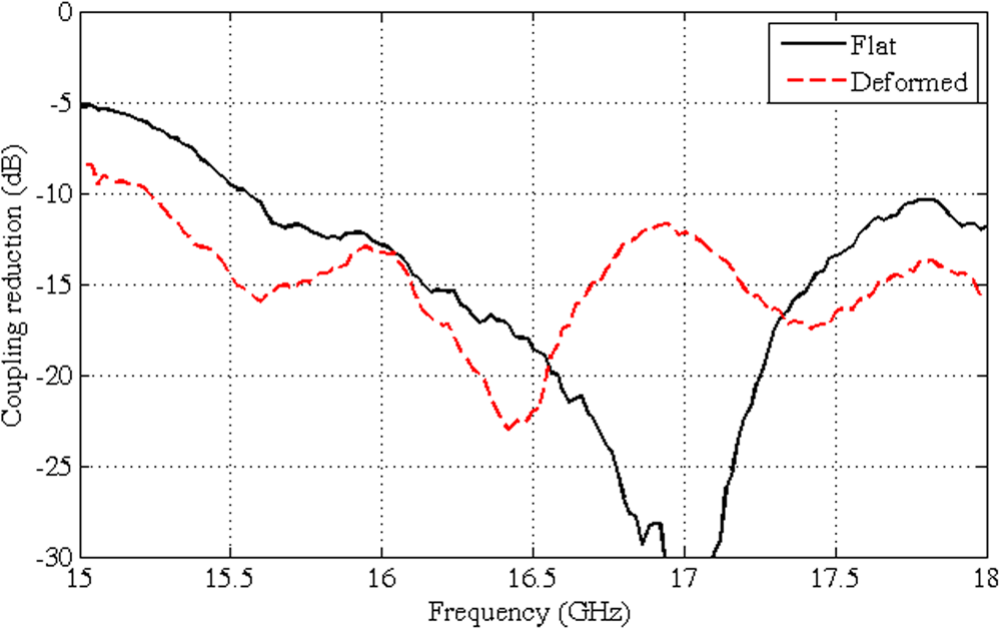
Measured reduction of the power received by a 15 dBi standard horn located in the specular direction (*θ *= 40°, *ϕ *= 90°, see Fig. 8) when the metasurface is present, either flat or deformed, with respect to the continuous metallic plate.

Figure 9 shows the amplitude map of the co-pol. and cross-pol. field acquired on the measurement plane for both the continuous metallic plate and the metasurface, when the MLI is flat. Also, Fig. 10 shows the co-polarization maps when the MLI is slightly deformed with respect to the flat ideal surface, as shown in Fig. 10c. In both the cases, we note that a reduction of about 15 dB of the maximum power density, with respect to the continuous metallic plate, is obtained when the metasurface is present. To better quantify this effect, Fig. 11 compares the reduction of power received by a 15 dBi standard horn, which is located in the specular direction, when the flat continuous metallic plate is replaced by the metasurface, either flat or deformed.

**Figure 12:**
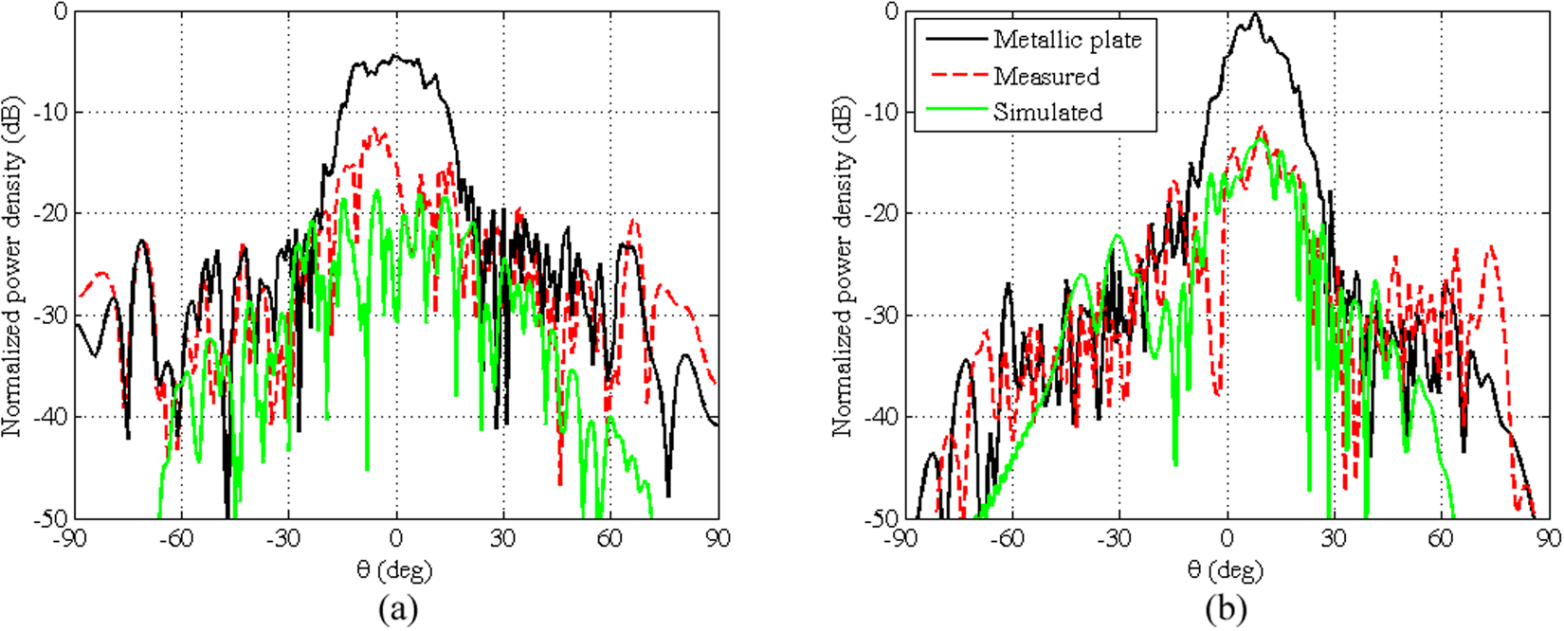
Normalized patterns in the principal planes of the co-pol. field scattered at 16.8 GHz by the continuous metallic plate (black) and by the manufactured metasurface either measured (red) or simulated (green): (**a**) *ϕ *= 0° and (**b**) *ϕ* = 90°.

**Figure 13:**
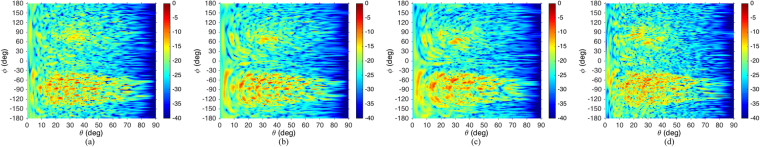
Power density distribution at 16.6 GHz in the whole space in presence of the modulated metasurface for the reference scenario of Fig. 1 when the feeding horn is rotated by (**a**) 20°, (**b**) 40°, (**c**) 60°, (**d**) 80° along the azimuthal coordinate.

**Figure 14:**
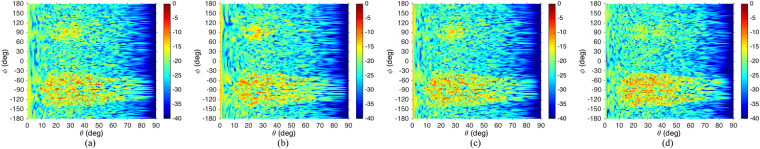
Power density distribution at 16.6 GHz in the whole space in presence of the modulated metasurface for the reference scenario of Fig. 1 when the feeding horn is (**a**) 0°, (**b**) 20°, (**c**) 60°, (**d**) 80° tilted with respect to the horizon.

Figure 12 shows the measured radiation patterns of the field scattered by the continuous metallic plate and by the manufactured metasurface. In Fig. 12, the simulated pattern is also shown in the case of a 35μm under-etching of the patches, which has been identified *a posteriori* by a microscope inspection on the manufactured prototype. This slightly reduced dimension of the patches suggested us to perform both the measurement and the numerical analysis to the higher frequency of 16.8 GHz to which the original design is theoretically shifted. The simulated and the measured values have been normalized in such a way that their maxima match when the continuous metallic plate is analyzed. The good conformity confirms the capacity of predicting in a reliable way the metasurface effect. This allows us to be confident that the results shown in Figs 13 and 14, relevant to the 1.7 × 2.3 m^2^ metasurface (i.e. the modulated metasurface for the reference scenario of Fig. 1), are valid. These figures show the power density, normalized to the maximum power density relevant to the flat continuous metallic plate, scattered by the metasurface in the whole space when the square frame element has been used, for different azimuthal and elevation incidence angles. It is worth noting that, irrespective of the incidence angle, in the forward region we obtain a power reduction better than 10 dB with respect to the continuous metallic plate case. In the backward region we can note an increase of the power density since the scattered field is distributed almost uniformly in the whole space. The phase of the scattered field, not shown here for sake of brevity, has similar distribution of the nominal one shown in Fig. 5d.

**Figure 15:**
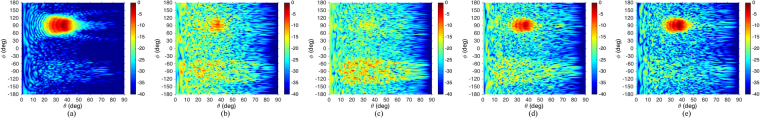
Power density distribution in the whole space in presence of the modulated metasurface for the reference scenario of Fig. 1 when the working frequency is (**a**) 14.6 GHz, (**b**) 15.6 GHz, (**c**) 16.6 GHz, (**d**) 17.6 GHz, (**e**) 18.6 GHz.

Regarding the flat metasurface case, Fig. 15 shows the power density distribution versus frequency. As expected, the reduction effectiveness of more than 15 dB is limited to about 1 GHz, since the intrinsic resonance of the metasurface. However, it can be noted that outside the working band the metasurface response tends to the one of the continuous metallic plate, thus not introducing any negative effect.

## Conclusion

The problem of the coupling between antennas, whose position is not exactly known with respect to an interference surface, which geometry is only approximately defined, has been addressed. A modulated metasurface can be used to reduce this problem. In this paper, it has been demonstrated that the actual field scattered by the metasurface when a completely random approach is exploited for its design, can not be predicted with a simple Physical Optics technique that makes use of the local periodicity approximation, as previously suggested by other authors. In fact, since the elements are sub-wavelength, the phase variation introduced by each element is strongly influenced by the dimensions of the adjacent ones. Therefore, fast variations of the phase along the metasurface, as required by a completely random approach, can not be realized. Here, it has been suggested to split up the metasurface in smaller areas of 5λ side, where the elements are designed to spread the scattered field with a broad axial-symmetric radiation pattern. However, a smooth phase variation between adjacent elements is maintained. Furthermore, the phase delay introduced by the elements of a certain area differs from the phase delay of the adjacent areas for a constant value. Such phase value is, however, chosen with a pseudo random scheme that allows a smooth phase variation between adjacent areas. The comparison of the expected scattered field with the results of a full-wave simulation, and the measurements, proves both the reliability of the proposed design approach, and the effectiveness of the modulated metasurface in reducing the antennas coupling of more than 15 dB. A insensitivity both from the antennas position with respect to the metasurface, and from a small metasurface deformation has also been proven.
